# Correction: Self-adaptive multiscaling algorithm for efficient simulations of many-protein systems in crowded conditions

**DOI:** 10.1039/c8cp91925a

**Published:** 2018-12-05

**Authors:** Sergio A. Hassan

**Affiliations:** Center for Molecular Modeling, OIR/CIT, National Institutes of Health, U.S. DHHS, USA.

Correction for ‘Self-adaptive multiscaling algorithm for efficient simulations of many-protein systems in crowded conditions’ by Sergio A. Hassan *et al., Phys. Chem. Chem. Phys.*, 2018, DOI: 10.1039/c8cp05517c.

The author would like to correct eqn (13)–(15) in the published article. $M_{i}$ should be amended to M in the upper limit of the second sum symbols as shown in the corrected equations below.

(13) $$R_{p}(r)\approx R_{0,p}+\overset{N_{I,0}}{\underset{\begin{array}{c} q\in I\\ q\neq p \end{array}}{\sum }}c_{pq}\text{exp}\left(-B_{p}r_{pq}\right)+\overset{M}{\underset{K\neq I}{\sum }}\overset{N_{K,\lambda_{K}}}{\underset{q\in K}{\sum }}c_{pq}\text{exp}\left(-B_{p}r_{pq}\right)$$

(14) $$A_{p}(r)\approx A_{0,p}-\overset{N_{I,0}}{\underset{\begin{array}{c} q\in I\\ q\neq p \end{array}}{\sum }}c_{pq}^{\prime }\text{exp}\left(-B_{p}^{\prime }r_{pq}\right)-\overset{M}{\underset{K\neq I}{\sum }}\overset{N_{K,\lambda_{K}}}{\underset{q\in K}{\sum }}c_{pq}^{\prime }\text{exp}\left(-B_{p}^{\prime }r_{pq}\right)$$

(15) $$\alpha_{p}(r)\approx \alpha_{0,p}-\overset{N_{I,\lambda_{I}}}{\underset{\begin{array}{c} q\in I\\ q\neq p \end{array}}{\sum }}c_{pq}^{\prime \prime }\text{exp}\left(-B_{p}^{\prime \prime }r_{pq}\right)-\overset{M}{\underset{K\neq I}{\sum }}\overset{N_{K,\lambda_{K}}}{\underset{q\in K}{\sum }}c_{pq}^{\prime \prime }\text{exp}\left(-B_{p}^{\prime \prime }r_{pq}\right)$$

The Royal Society of Chemistry apologises for these errors and any consequent inconvenience to authors and readers.

